# Does vitamin D reduce the mortality rate of *Plasmodium* infection?: a systematic review and meta-analysis

**DOI:** 10.1186/s12936-023-04612-4

**Published:** 2023-06-05

**Authors:** Narges Kalantari, Mahdi Sepidarkish, Salman Ghaffari, Sahar Rostami-Mansoor

**Affiliations:** 1 Cellular and Molecular Biology Research Center, Health Research Institute, Babol University of Medical Sciences, Babol, Iran; 2 Department of Biostatistics and Epidemiology, School of Public Health, Babol University of Medical Sciences, Babol, Iran; 3 Department of Mycology and Parasitology, School of Medicine, Babol University of Medical Sciences, Babol, Iran

**Keywords:** Malaria, *Plasmodium*, Survival rate, Vitamin D

## Abstract

**Background:**

Vitamin D supplementation is recommended as an effective adjunct to counteract malaria pathogenesis, but the evidence on this point is limited and controversial. This systematic review and meta-analysis aimed to investigate the effect of vitamin D administration on the survival rate of *Plasmodium*-infected animals in experimentally-induced malaria on days 6 and 10 post-infection.

**Methods:**

Five electronic databases were searched up to 20 December 2021. The pooled risks ratio (RR) and associated 95% confidence interval were estimated using the Restricted-maximum likelihood (REML) random-effects model. Heterogeneity was assessed by Cochran’s Q test and I^2^ value. Sub-group analyses were used to identify the sources of heterogeneity for several variables, such as type of vitamin D, type of intervention, and dose of vitamin D.

**Results:**

Out of 248 articles found in the electronic database, six were eligible for inclusion in the meta-analysis. The current study found that the pooled random effect of risks ratio favored a statistically significant effect of vitamin D administration on survival rate in infected mice on day 6 post *Plasmodium* infection (RR = 1.08, 95%CI 1.03, 1.15, p < 0.99; I^2^ = 0%). It also found that vitamin D administration significantly affected the survival rate on day 10 post-infection (RR = 1.94, 95%CI 1.39, 2.71, p < 0.001; I^2^ = 69.02%). Subgroup analyses demonstrated a significant pooled RRs of the positive effect of vitamin D administration for cholecalciferol (RR = 3.11, 95%CI 2.41, 4.03, p < 0.001; I^2^ = 0%), doses higher than 50 µg/kg (RR = 3.37, 95%CI 2.55, 4.27, p < 0.001; I^2^ = 0%), and oral administration (RR = 3.01, 95%CI 2.37, 3.82, p < 0.001; I^2^ = 0%).

**Conclusion:**

This systematic review and meta-analysis showed that vitamin D administration positively affects the survival rate in *Plasmodium*-infected mice. Since, the mouse model may not accurately reproduce the clinical and pathological features of human malaria, future research should investigate the impact of vitamin D in human malaria.

**Supplementary Information:**

The online version contains supplementary material available at 10.1186/s12936-023-04612-4.

## Background

Malaria is a parasitic disease that primarily affects impoverished tropical and sub-tropical regions of the world. It is a significant cause of mortality, particularly among pregnant women and children under five in endemic areas like sub-Saharan Africa [1]. The World Health Organization (WHO) reported that nearly half of the world's population is at risk of contracting malaria [2]. Six species of *Plasmodium* are known to infect humans, all of which cause anaemia. However, severe malaria is caused by *Plasmodium falciparum* and *Plasmodium vivax* infections, with the former species causing the most severe form of the disease, including cerebral malaria (CM) and severe anaemia, which is a leading cause of death in children under five [3]. Despite extensive research and the use of highly effective anti-malarial drugs, malaria still has high morbidity and mortality rates. Unfortunately, global efforts to develop a highly effective vaccine against malaria have not yet been successful [4]. The search for additional therapies to supplement anti-malarial treatments is critical due to the high morbidity and mortality rates of malaria. Vitamin D supplementation has been suggested as a potential therapy to reduce the burden of malaria [3]. Some studies have reported a possible effect of vitamin D in reducing the morbidity and mortality rates of malaria in animal models [3].

Vitamin D is a fat-soluble vitamin that is obtained endogenously through solar ultraviolet B radiation on the skin or exogenously through the diet [5]. It is an essential vitamin for the proper functioning of the human and animal bodies. Vitamin D is crucial in regulating cell growth, inhibiting inflammation, and supporting immune system function. It reduces the release of pro-inflammatory cytokines from T-helper (Th)-1 and Th17 cells, including tumour necrosis factor (TNF), interleukin-2 (IL-2), and interferon-gamma (IFN-γ), while increasing the secretion of anti-inflammatory factors, such as IL-4, from Th2 cells [6].

However, growing evidence indicates that vitamin D can decrease the intensity of viral infections by inducing antimicrobial peptides [7] or inhibiting microorganism proliferation by inducing nitric oxide synthase (NOS) activity and NO accumulation [8]. Concerning *Plasmodium* infection, documented reports show that vitamin D supplementation has reduced malaria morbidity and mortality rates [5]. Moreover, Dwivedi et al*.* reported that combining an anti-malarial drug (arteether) with vitamin D resulted in a greater improvement in the outcome of experimental CM than using arteether alone [9]. They demonstrated that vitamin D improves the integrity of the blood–brain barrier (BBB) and increases survival in mice infected with *Plasmodium berghei* [9].

However, these studies were conducted in mouse models, which have not fully replicated the clinical and pathophysiological features of severe malaria in humans. Nevertheless, animal models are essential to evaluate potential interventions before they are tested in humans [10]. Therefore, this systematic review and meta-analysis aims to investigate the effect of vitamin D administration on the survival rate of animals infected with *Plasmodium* species in experimentally-induced malaria.

## Methods

### Literature eligibility

This systematic review and meta-analysis investigates the effect of vitamin D on the survival rate of experimentally *Plasmodium* infected animals. It was carried out according to PRISMA guidelines as a preferred and reliable protocol [11]. The eligibility criteria in this systematic review and meta-analysis were as follows: (1) The original peer-reviewed studies, (2) The experimental model of malaria that infected by any species of *Plasmodium* and treated by vitamin D, (3) The studies included the control group as vehicle receiver, and (4) The literature that measured the effect of vitamin D on the mortality rate or survival rate of the infected animal. This study did not restrict to the time of publication. Exclusion criteria were as follows: (1) human studies, (2) reviews, (3) conference papers, (4) duplicate publications, and (5) lack of appropriate controls.

### Literature search and study selection

Searching was carried out using five electronic databases (PubMed, Web of Science, Scopus, Cochrane and ProQuest) up to 20 December 2021. The main search terms were “malaria” and “vitamin D, and the generic syntax for the PubMed database was as follows (Malaria OR Paludism OR “Marsh Fever” OR “Remittent Fever” OR “Plasmodium Infections” OR “Plasmodium Infection” OR (Infections AND Plasmodium) OR (Infection AND Plasmodium(( AND (Vitamin D OR Ercalcidiol OR Cholecalciferol OR Ergocalciferols OR Dihydrotachysterol OR “25-Hydroxycalciferol” OR “25 Hydroxycalciferol” OR “25-Hydroxycalciferol” OR “25-Hydroxyvitamin D2” OR “25 Hydroxyvitamin D 2” OR “25-Hydroxyvitamin D 2” OR “25-Hydroxycalciferol” OR “9,10-Secoergosta-5,7,10(19),22-tetraene-3 beta,25-diol”).

All articles from the electronic databases were imported to Endnote (ver. 7) and then duplicate publications were removed. Afterward, two independent researchers (N.K and S.R-M) initially screened articles based on the title and abstract information. Additionally the references of relevant studies were checked to find potentially related articles.

### Data extraction

The selected articles were reviewed in full text and the studies which accomplished the criteria above were included in a data extraction form in an Excel sheet by two independent reviewers (N.K and S.R-M). Any disagreement between them was resolved by consulted with a third reviewer (S.Gh). The data was taken out from the included studies and arranged for the following variables: author last name, year of publication, number of mice in case and control groups, mice bread, parasite species, number of injected parasites, time of parasite injection, type of intervention, type of vitamin D, the dose of vitamin D, number of alive mice in case and control groups 6 days after infection and number of alive mice in both case and control groups 10 days post-infection.

### Data synthesis and statistical analysis

The effect size for survival rate was measured as the risk ratios (RRs) with corresponding 95% confidence intervals (CIs) obtained by Mantel-Hansel method. Data were combined using the random-effects model of Restricted-maximum likelihood (REML) method for variance estimation. Heterogeneity of the studies was assessed graphically with forest plots and statistically by chi-square-based Q statistic and I^2^ value. Heterogeneity was considered significant at a P-value of < 0.10 in Q-test or I^2^ > 40%. The sub-group analysis was implemented to identify the source of heterogeneity for parasite species, mice breed, mice age on administration day, the number of injected parasites, time of vitamin D administration (before/after parasite injection), type of vitamin D, type of intervention and dose of vitamin D. Publication bias was assessed by visually inspecting the funnel plot (SE plotted vs. log RR) and statistically by conducting the Egger's regression test to detect asymmetry of the plot. Results of publication bias were further validated by constructing trim and fill counter-enhanced funnel plot. Statistical analyses were performed using Stata software (Version 17.0) (Stata Corp, College Station, Texas) and Review Manager software (v 5.4; Cochrane Collaboration).

### Study quality assessment

The study quality assessment was evaluated by two independent authors according to the risk of bias (RoB) tool of SYRCLE (Systematic Review Centre for Laboratory Animal Experimentation [15]. All references were evaluated according to ten methodological domains. The answer "yes" for each item indicated low risk of bias, the answer "no" showed high the risk of bias and "unclear" demonstrated that the item was not reported and, therefore, the risk of bias was unknown.

## Results

### Features of the included studies

**Fig. 1:**
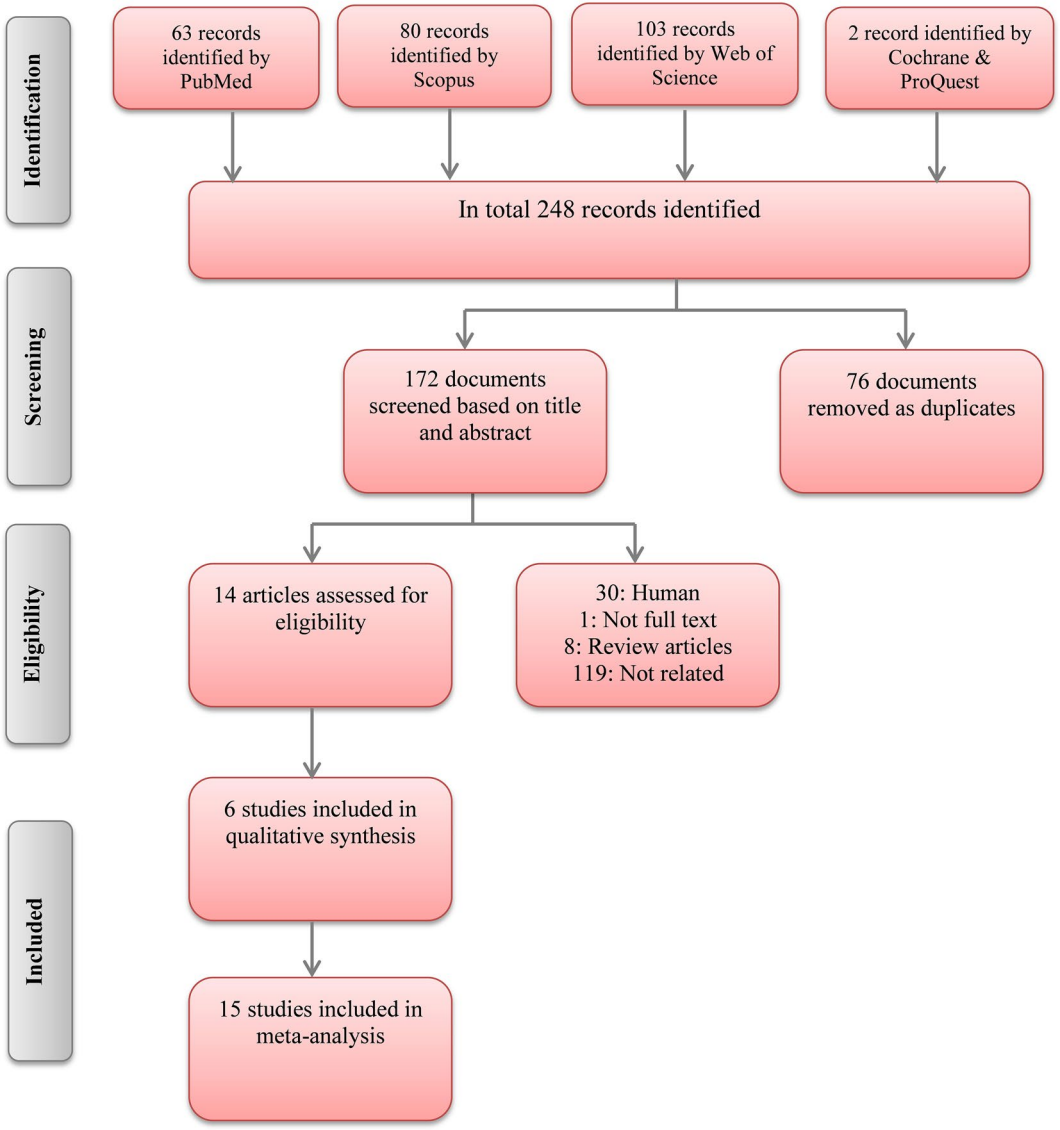
PRISMA flowchart of study identification and selection

In total 248 articles were found by systematic search in the present study. After eliminating duplicate studies, 172 articles remained. One-hundred fifty-eight unrelated studies were removed based on the title and abstract. The full-text of 14 studies were evaluated based on inclusion and exclusion criteria. Finally, 6 studies were accounted eligible for quality assessment. Among 6 included studies, 3 studies evaluated the effect of vitamin D on different strains of mice, different doses of vitamin D, and different times of vitamin D administration. These articles were considered as separate studies and, therefore, 15 datasets were included in meta-analysis. The process of study selection is summarized in PRISMA flowchart (Fig. 1).

The main characteristics of selected articles are described in Table 1. The publication date of the articles was from 2011 to 2019. Five strains of mice including C57BL/6 (n = 135), C57BL/10 (n = 5), BALBC (n = 28), IFNγ -/- (n = 10), vitamin D receptor (VDR) -/- (n = 5) were used. Mice were inoculated by 1 × 10^4^–3 × 10^6^ infected red blood cells (RBC) from two different species of *Plasmodium* (*Plasmodium berghei* and *Plasmodium chabaudi*). Animals included in case group received 0.5–50 (μg/kg) vitamin D (cholecalciferol or 1,25 (OH)_2_ D3) through intramuscular (IM) injection, intraperitoneal (IP) administration, sub cutaneous (SC) delivery and oral gavage during 3–7 days. Finally, the number of alive mice was assessed 6 and 10 days after infection.

**Table 1:** Summary of studies reporting the relationship between vitamin D administration and mortality rate in experimental model for malaria

Author	No. case	No. control	Mice breed	Parasite species	No. Parasites	Type of intervention	Time of vitamin administration*	Type of vitamin D	Dose of vitamin (μg/kg)	Alive case (day 6)	Alive control (day 6)	Alive case (day 10)	Alive control (day 10)
Dwivedi et al. [9]	9	18	C57BL/6	*P. berghei*	3 × 10^6^	IM	After	1,25 (OH)_2_ D3	50	8	15	3	0
He et al. [6]	28	40	C57BL/6	*P. berghei*	1 × 10^6^	Oral	Before	Cholecalciferol	50	28	36	28	12
He et al. [6]	23	40	C57BL/6	*P. berghei*	1 × 10^6^	Oral	After	Cholecalciferol	50	23	36	23	12
He et al. [6]	40	40	C57BL/6	*P. berghei*	1 × 10^6^	Oral	After	Cholecalciferol	50	40	36	40	12
He et al. [6]	10	10	C57BL/6	*P. berghei*	1 × 10^6^	IP	Before	Cholecalciferol	0.5	10	9	2	3
Waisberg et al. [5]	10	10	C57BL/6	*P. berghei*	1 × 10^6^	IP	Before	Cholecalciferol	0.5	6	5	0	0
Wu et al., 2018 [3]	10	10	C57BL/6	*P. berghei*	1 × 10^6^	Oral	Before	Cholecalciferol	50	10	9	10	2
Yamamoto et al. [14]	6	6	BALBC	*P. chabaudi*	141 × 10^4^	SC	After	1,25 (OH)_2_ D3	3	5	6	2	1
Yamamoto et al. [14]	5	5	C57BL/6	*P. chabaudi*	141 × 10^4^	SC	NA	1,25 (OH)_2_ D3	3	5	5	5	5
Yamamoto et al. [14]	5	5	C57BL/10	*P. chabaudi*	141 × 10^4^	SC	After	1,25 (OH)_2_ D3	3	5	5	5	5
Yamamoto et al. [14]	5	5	IFNγ -/-	*P. chabaudi*	1 × 10^4^	SC	After	1,25 (OH)_2_ D3	3	5	5	3	0
Yamamoto et al. [14]	5	5	IFNγ -/-	*P. chabaudi*	1 × 10^4^	SC	After	1,25 (OH)_2_ D3	3	5	5	3	0
Yamamoto et al. [14]	5	5	VDR -/-	*P. chabaudi*	1 × 10^4^	SC	Before	1,25 (OH)_2_ D3	3	5	5	5	5
Yamamoto et al. [8]	6	6	BALBC	*P. chabaudi*	141 × 10^4^	SC	After	1,25 (OH)_2_ D3	0.6	6	6	6	2
Yamamoto et al. [8]	16	10	BALBC	*P. chabaudi*	141 × 10^4^	Oral	Before	1,25 (OH)_2_ D3	0.6	16	8	10	5

^a^After or before infection

### Main analysis

**Fig. 2:**
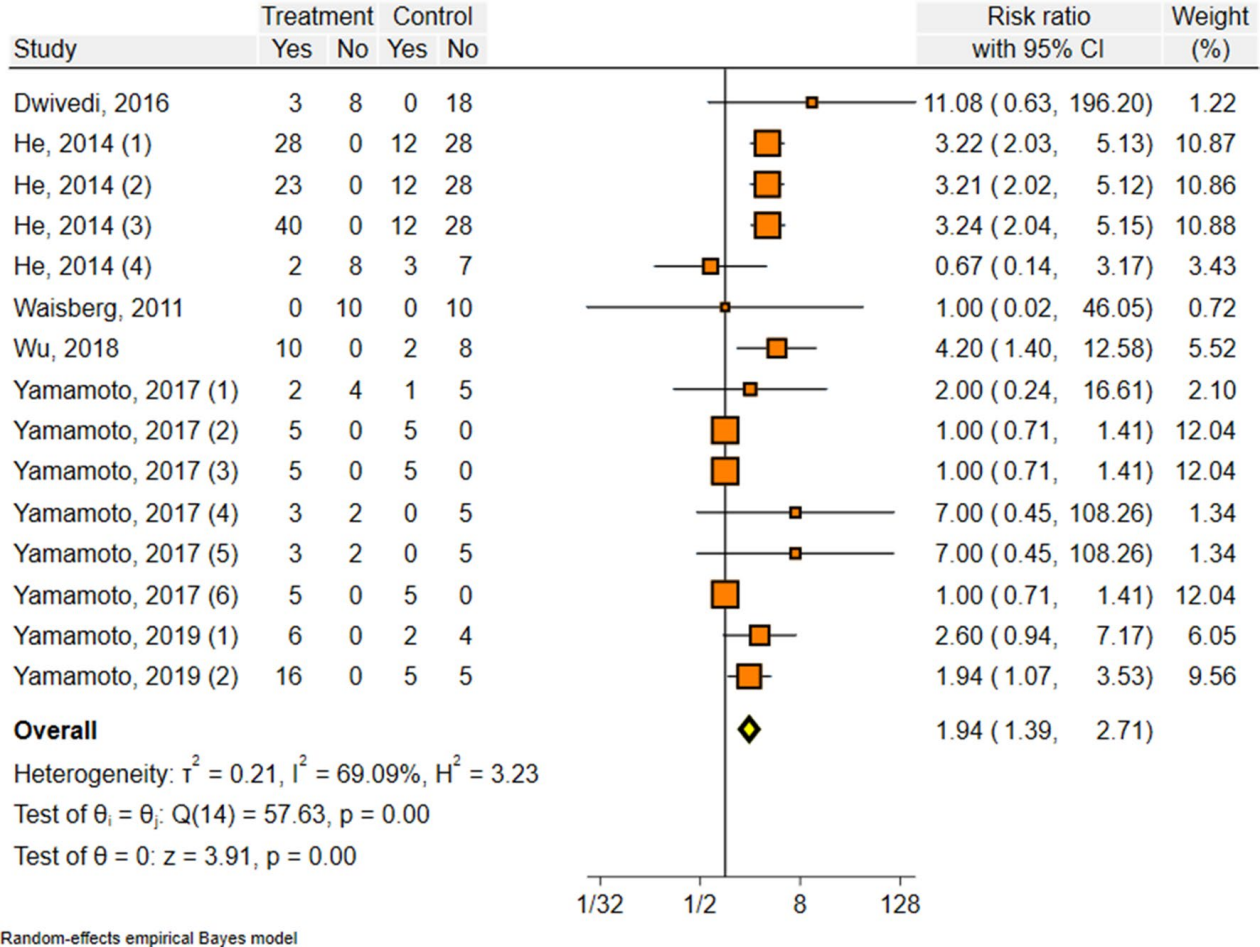
Forest plot displaying the effect of vitamin D administration on survival rate, 10 days after infection by *Plasmodium* spp. RR > 1 shows a positive effect of vitamin D to survive animals. Point estimates and 95% CI are shown for pooled results and individuals

**Fig. 3:**
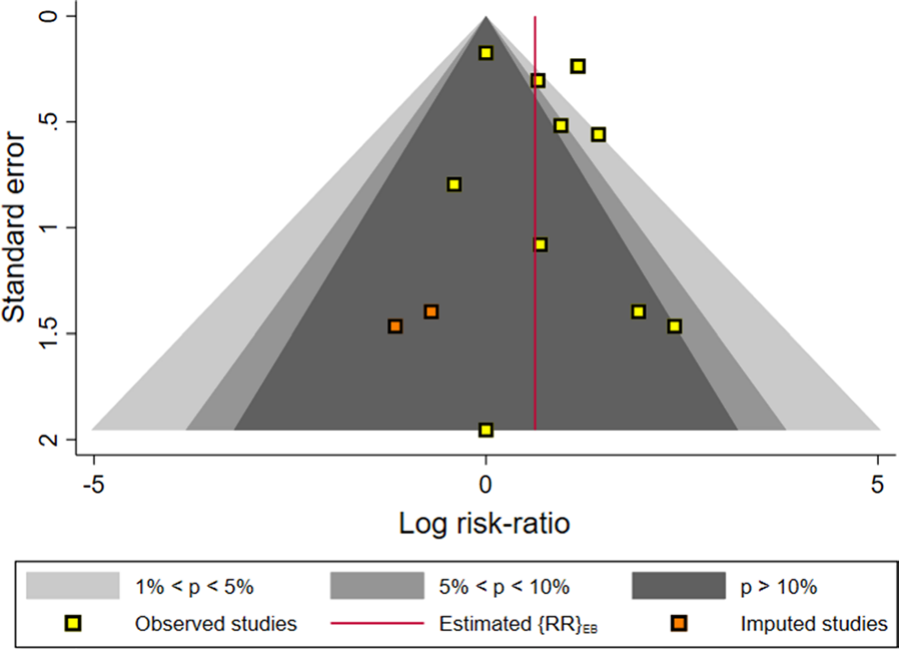
Funnel plot of standard error by log risk ratio from the studies on the effect of vitamin D administration on survival rate, 10 days after infection by *Plasmodium* spp

Overall, the pooled random effect favored a significant effect of vitamin D administration on mice survival rate on day 6 after *Plasmodium* infection (RR = 1.08, 95%CI 1.03, 1.15, p < 0.001) with completely low heterogeneity in studies (I^2^ = 0.00%, P = 0.99) (Table 2 and Additional file 1: Fig S1). Furthermore, on day 10 post *Plasmodium* infection, the pooled random effect of risk ratio showed a statistically significant effect of vitamin D administration on survival rate in infected mice (RR = 1.94, 95%CI 1.39, 2.71, p < 0.001; I^2^ = 69.02%) (Table 3 and Fig. 2). In subgroup analyses, significant pooled RRs of positive effect of vitamin D administration were seen for doses higher than 50 µg/kg (RR = 1.10, 95%CI 1.03, 1.18, p < 0.001) on day 6 and [RR = 3.37 (95% CI 2.55–4.27)] on day 10. Similarly, analysis of the type of vitamin D showed that cholecalciferol has a significant positive effect on survival chance (RR = 1.10, 95%CI 1.03, 1.17) on day 6 and (RR = 3.11, 95%CI 2.41, 4.03) on day 10. Oral administration of vitamin D showed a significant positive effect on the survival rate in *Plasmodium* infected mice (RR = 1.11, 95%CI 1.04, 1.18) on day 6 and (RR = 3.01, 95%CI 2.37, 3.82) on day 10. Interestingly, the time of vitamin D administration showed that the use of this supplement before parasitic injection had more positive effect on the survival rate on day 6 (RR = 1.11, 95%CI 1.01, 1.22), while the survival rate on day 10 were higher in the groups of mice that received vitamin D after parasite infection (RR = 2.05, 95%CI 1.30, 3.23). The results obtained from sub-groups analysis of other variables are also presented in Tables 2, 3. The evaluation of the funnel plots suggests the presence of publication bias; i.e. empty quadrant in which potentially small unpublished studies may have shown less survival risk reduction (Fig. 3 and Additional file: 2: Fig. S2).

**Table 2:** Sub-group analysis between vitamin D administration and mortality rate in experimental model of malaria (day 6)

Sub-group	No. studies	RR (CI 95%)	*I*^*2*^	p-value^a^
Parasite species				
*P. berghei*	7	1.10 (1.04,1.17)	0	0.999
*P. chabaudi*	8	1.02 (0.90, 1.015)	0	0.941
Mice breed				
C57/BL6	8	1.10 (1.03, 1.17)	0	0.999
Other	7	1.02 (0.88, 1.16)	0	0.891
Mice age in administration day				
< 49 days	6	1.10 (1.04, 1.17)	0	0.999
> 60 days	9	1.02 (0.91, 1.15)	0	0.964
Number parasites				
≤ 10^6^	9	1.09 (1.03, 1.16)	0	0.999
> 10^6^	6	1.04 (0.90, 1.19)	0	0.807
Dose of vitamin D				
= 50 μg/kg	5	1.10 (1.03,1.18)	0	0.999
< 50 μg/kg	10	1.04 (0.94, 1.15)	0	0.994
Time of vitamin administration				
Before infection	6	1.11 (1.01, 1.22)	0	0.967
After infection	9	1.07 (1.10, 1.15)	0	0.961
Type of intervention				
Oral	5	1.11 (1.04, 1.18)	0	0.967
Injection	10	1.02 (0.91, 1.13)	0	0.998
Type of vitamin D				
Cholecalciferol	6	1.10 (1.03, 1.17)	0	0.999
1,25 (OH)_2_ D3	9	1.02 (0.91, 1.14)	0	0.967
All studies	15	1.08 (1.03, 1.15)	0	0.996

^a^Test for heterogeneity

**Table 3:** Sub-group analysis between vitamin D administration and mortality rate in experimental model of malaria (day 10)

Sub-group	No. studies	RR (CI 95%)	*I*^*2*^	p-value^a^
Parasite species				
*P. berghei*	7	3.15 (2.44, 4.07)	0	0.521
*P. chabaudi*	8	1.28 (0.92, 1.77	50.79	0.143
Mice breed				
C57/BL6	8	2.36 (1.46, 3.83)	72.13	< 0.001
Other	7	1.40 (0.95, 2.07)	42.07	0.113
Mice age in administration day				
< 49 days	6	3.17 (2.45, 4.10)	0	0.436
> 60 days	9	1.23 (0.93, 1.63)	35.60	0.207
Number parasites				
≤ 10^6^	9	2.40 (1.55, 3.73)	64.66	0.001
> 10^6^	6	1.39 (0.91, 2.11)	56.01	0.095
Dose of vitamin D				
≥ 50 μg/kg	5	3.37 (2.55, 4.27)	0	0.925
< 50 μg/kg	10	1.19 (0.92, 1.54)	27.67	0.254
Time of vitamin administration				
Before infection	6	1.83 (1.08, 3.14)	67.27	0.001
After infection	9	2.05 (1.30, 3.23)	70.44	< 0.001
Type of intervention				
Oral	5	3.01 (2.37, 3.82)	0	0.612
Injection	10	1.53 (0.82, 1.56)	32.04	0.332
Type of vitamin D				
Cholecalciferol	6	3.11 (2.41, 4.03)	0	0.448
1,25 (OH)_2_ D3	9	1.28 (0.95, 1.72)	41.08	0.101
All studies	15	1.94 (1.39, 2.71)	0	< 0.001

^a^Test for heterogeneity

### Quality assessment

**Fig. 4:**
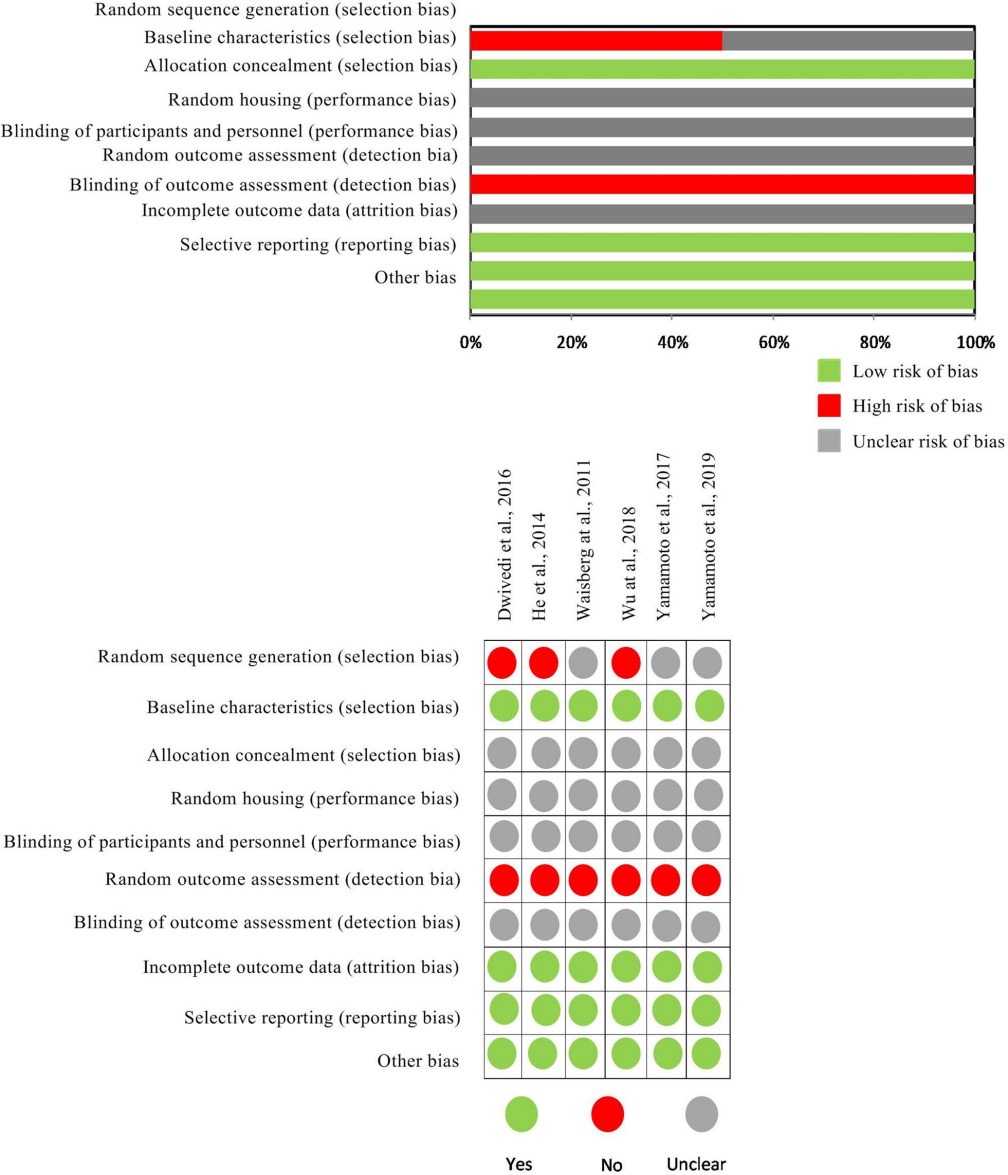
The risk of bias (ROB) assessment using SYRCLE´s tool. The percentage (%) shows the risk of bias for each methodological domain of the tool

Quality assessment was reported for overall methodological qualities for each included study (Fig. 4). None of the studies met all the methodological assumptions. Sixty-five percent (65%) of the studies did not describe animal randomization and 45% did not randomized animals. All the included studies balanced the basic characteristics (age, weight, strain, and sex), but none of them mentioned about allocation concealment, random housing, blinding participants and blinding of outcome assessment. However, all the studies presented adequate explanation about animals included in the analysis, reporting bias and other problems.

## Discussion

Malaria prevention and control are urgent public health issues, and discovering new tools for prevention and therapy is crucial. Previous research on animal models has indicated that vitamin D may impact malaria pathogenesis [5, 6]. Given the limited human evidence in this field, a systematic review and meta-analysis of animal literature was conducted to investigate the potential effect of vitamin D administration on the survival rate of *Plasmodium*-infected mice. This study showed that vitamin D administration improves survival chances on days 6 and 10 after infection. The most significant difference in survival rate between the case and control groups was observed on day 10. Some studies (He et al. [6]; Wu et al*.* [3]; Yamamoto et al*.* [8]) reported a significant effect of vitamin D administration on the survival chance of *Plasmodium* infected mice (Fig. 2). Three out of four studies that used *P. berghei* demonstrated that vitamin D protects against CM in experimentally infected mice [3, 6, 9]. Among the included studies, Waisberg et al*.* reported that mice receiving vitamin D had better clinical scores and higher haemoglobin levels without significantly reducing the mortality rate [5]. However, previous studies have shown that *Plasmodium* infection in mice can lead to hyperinflammation, intravascular leukocyte accumulation, and BBB leakage, all of which are common causes of death in young children and mice [12]. Therefore, developing strategies to stimulate proper immune responses to *Plasmodium* spp. is essential to reducing the mortality rate [3]. In this regard, documented evidence has shown that vitamin D can decrease malaria symptoms by modifying the immune system in infected mice. These modifications may occur through reducing the expression of cytokines, such as IFN-γ and TNF, ameliorating the infiltration of inflammatory cells into the brain, increasing vascular integrity, and inducing NO production [3, 6, 8, 13, 14]. In human malaria, Cusik et al*.* reported low vitamin D levels in children with severe anaemia and CM [15]. However, other studies have shown no association between malaria infection and vitamin D status [16].

Vitamin D has two distinct mechanisms of action to diminish parasites: genomic and non-genomic. The genomic mechanism involves vitamin D indirectly protecting patients by regulating the immune system through binding to intracellular receptors that modulate gene expression. The non-genomic mechanism of vitamin D, on the other hand, is characterized by its ability to directly kill parasites. Previous studies on humans have shown that administering vitamin D can improve anaemia by increasing the proliferation of erythroid progenitor cells and reducing pro-inflammatory cytokines [17]. The findings obtained from present study demonstrated that cholecalciferol had a more positive effect on the survival rate of infected mice. This could be explained by the efficient conversion of cholecalciferol stored in adipose tissue and muscle into the active form of vitamin D (1,25 (OH)2 D3) [18]. Moreover, this study showed a greater RR for the effect of vitamin D on the survival rates of mice infected with *P. berghei*. It is well documented that *P. berghei* is a lethal form of *Plasmodium* that induces CM in C57BL/6 mice, while *P. chabaudi* elicits partial mortality in C57BL/6 and C57BL/10 mice [19]. Therefore, the differences between the case and control were less significant in mice infected with *P. chabaudi*.

The present study observed moderate heterogeneity (I^2^ = 69.09%, P = 0.001). The literature reports several sources of heterogeneity in animal studies, including differences in the species, age, and sex of animals used, as well as variations in study design and methods. Subgroup analyses were performed to identify potential sources of heterogeneity. The results showed that the type of intervention, parasite species, age and breed of mice, number of injected parasites, type and dose of vitamin D, and timing of administration might be specific causes of heterogeneity.

Some limitations in the present study could have affected the results of the current meta-analysis. These limitations are as follows: (i) the limited number of primary studies, which may have affected the power of the results; (ii) the protective effect of vitamin D supplementation was reported by only five data sets from three laboratories; (iii) this study only examined the effects of this vitamin on the outcome of infection in mice and cannot be generalized to humans; (iv) the studies' quality was unacceptable, especially regarding randomization and blinding assessment; (v) one potentially relevant study via this systematic review was identified, but the full text was not available which increasing the risk of missing eligible data; (vi) the study search was limited to articles published in English, increasing the risk of missing some eligible studies.

## Conclusion

This systematic review and meta-analysis demonstrated that the administration of vitamin D positively affects the survival rate of *Plasmodium*-infected mice. Although the mouse model may not accurately reproduce the clinical and pathophysiological features of human malaria, such research is essential for providing valuable information to design appropriate studies in humans. However, the current study is the first systematic review and meta-analysis to address the effects of vitamin D on *Plasmodium* infection. Further studies should be carried out to investigate the impact of vitamin D on malaria in the future.

## Supplementary Information

**Additional file 1: Figure S1.** Forest plot displaying the effect of vitamin D administration on survival rate, 6 days after infection by *Plasmodium spp*. RR> 1 shows a positive effect of vitamin D to survive animals. Point estimates and 95% CI are shown for pooled results and individuals.

**Additional file 2: Figure S2.** Funnel plot of standard error by log risk ratio from the studies on the effect of vitamin D administration on survival rate, 6 days after infection by *Plasmodium *spp.

## Data Availability

The datasets analysed during the current study are available from the corresponding author on reasonable request.

## Abbreviations

BBB: Blood brain barrier
CAMP: Cathelicidin antimicrobial peptide
CM: Cerebral malaria
CI: Confidence interval
CXCL9: Chemokine C-X-C motif ligand 9
CXCL10: Chemokine C-X-C motif ligand 10
IM: Intramuscular
IP: Intraperitoneal
IFN-γ: Interferon-gamma
IL-2: Interleukin-2
IL-4: Interleukin-4
NO: Nitric oxide
NOS: Nitric oxide synthase
RBC: Red blood cell
REML: Restricted-maximum likelihood
RoB: Risk of bias
RR: Risk ratio
SC: Subcutaneous
Th1: T-helper 1
Th2: T-helper 2
Th17: T-helper 17
TNF-α: Tumour necrosis factor alpha
VDR: Vitamin D receptor
WHO: World health organization
