# Supplementary figures and images for: Does vitamin D reduce the mortality rate of Plasmodium infection?: a systematic review and meta-analysis
Source: Malar J. 2023 Jun 5;22:173. doi: 10.1186/s12936-023-04612-4 (PMC10243038; doi:10.1186/s12936-023-04612-4)

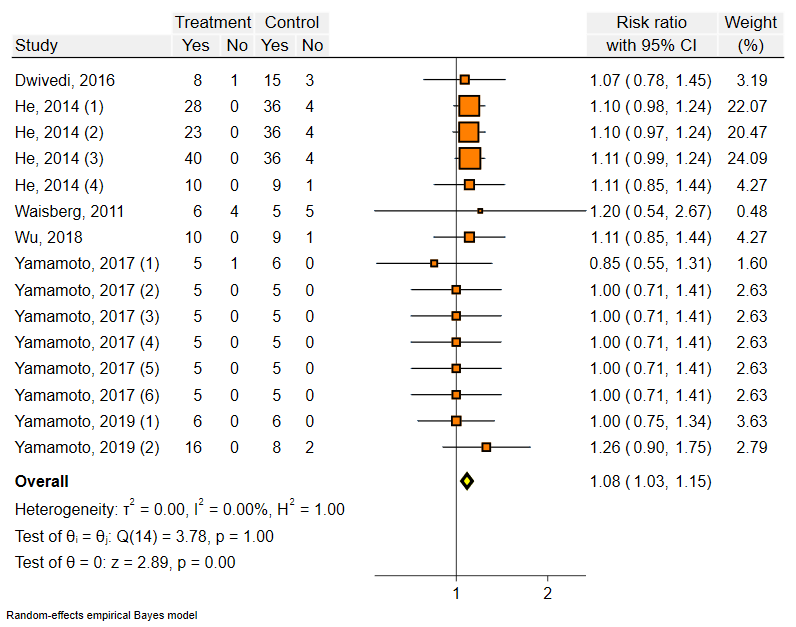

Supplement: Supplementary file 1 — Additional file 1: Figure S1. Forest plot displaying the effect of vitamin D administration on survival rate, 6 days after infection by Plasmodium spp. RR> 1 shows a positive effect of vitamin D to survive animals. Point estimates and 95% CI are shown for pooled results and individuals. [file 12936_2023_4612_MOESM1_ESM.tif]

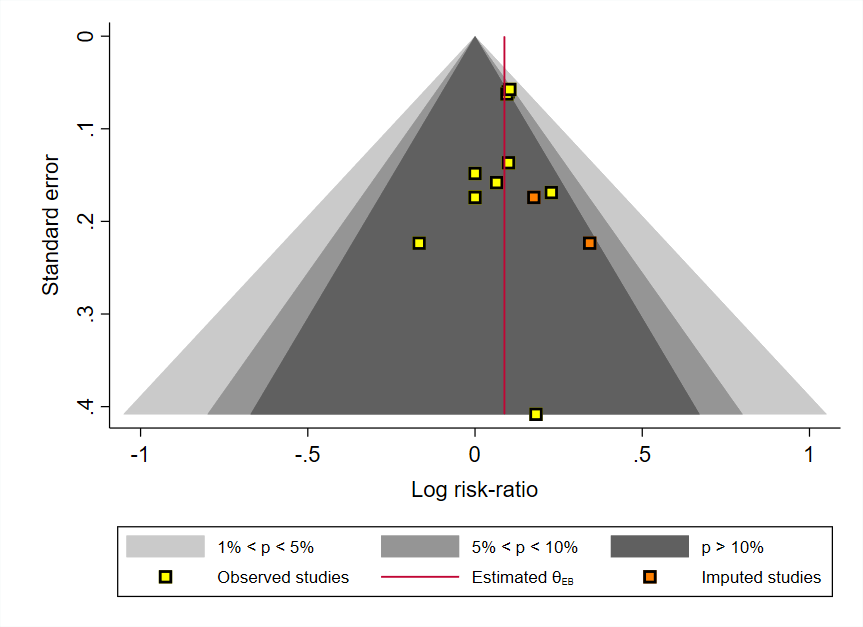

Supplement: Supplementary file 2 — Additional file 2: Figure S2. Funnel plot of standard error by log risk ratio from the studies on the effect of vitamin D administration on survival rate, 6 days after infection by Plasmodium spp. [file 12936_2023_4612_MOESM2_ESM.tif]
